# De novo aging-related NADPH diaphorase positive megaloneurites in the sacral spinal cord of aged dogs

**DOI:** 10.1038/s41598-023-49594-0

**Published:** 2023-12-14

**Authors:** Yinhua Li, Yunge Jia, Wei Hou, Zichun Wei, Xiaoxin Wen, Yu Tian, Lu Bai, Xinghang Wang, Tianyi Zhang, Anchen Guo, Guanghui Du, Zhuang Ma, Huibing Tan

**Affiliations:** 1 College of Physical Education and Sports Rehabilitation, Jinzhou Medical University, Jinzhou, 121001 Liaoning China; 2 Department of Anatomy, Jinzhou Medical University, Jinzhou, 121001 Liaoning China; 3 Key Laboratory of Neurodegenerative Diseases of Liaoning Province, Jinzhou Medical University, Jinzhou, 121001 Liaoning China; 4 Laboratory of Clinical Medicine Research, Beijing Tiantan Hospital, Capital Medical University, Beijing, 100050 China; 5 Department of Urology, Tongji Medical College Affiliated Tongji Hospital, Wuhan, 430030 Hubei China; 6 Department of Pathology, Heji Hospital Affiliated of Changzhi Medical College, Changzhi, 040611 Shanxi China; 7 Department of Neurology, Suizhou Central Hospital, Wuhan, 441300 China

**Keywords:** Diseases of the nervous system, Neural ageing

## Abstract

We investigated aging-related changes in nicotinamide adenine dinucleotide phosphate-diaphorase (NADPH-d) in the spinal cord of aged dogs. At all levels of the spinal cord examined, NADPH-d activities were observed in neurons and fibers in the superficial dorsal horn (DH), dorsal gray commissure (DGC) and around the central canal (CC). A significant number of NADPH-d positive macro-diameter fibers, termed megaloneurites, were discovered in the sacral spinal cord (S1–S3) segments of aged dogs. The distribution of megaloneurites was characterized from the dorsal root entry zone (DREZ) into the superficial dorsal horn, along the lateral collateral pathway (LCP) to the region of sacral parasympathetic nucleus (SPN), DGC and around the CC, but not in the cervical, thoracic and lumbar segments. Double staining of NADPH-d histochemistry and immunofluorescence showed that NADPH-d positive megaloneurites co-localized with vasoactive intestinal peptide (VIP) immunoreactivity. We believed that megaloneurites may in part represent visceral afferent projections to the SPN and/or DGC. The NADPH-d megaloneurites in the aged sacral spinal cord indicated some anomalous changes in the neurites, which might account for a disturbance in the aging pathway of the autonomic and sensory nerve in the pelvic visceral organs.

## Introduction

The caudal lumbar and sacral spinal cord is important for controlling the function of the large intestine, pelvic muscles and the urogenital organs^1–3^. The sacral spinal cord is specifically related to the control of the intestine, bladder and sexual dysfunction^4–6^. In the dorsal gray commissure (DGC) in the S1–S3 segments of the cat spinal cord, this region receives the terminals of visceral afferent fibers in the pelvic nerves and somatic afferent fibers for the pudendal nerves through the Lissauer's tract (LT) and its lateral- and medial-collateral projections^7–10^. The retrograde transganglionic labeling of primary afferent fibers from the bladder^11^, urethra^12^ and external urethral sphincter^13^, as well as from the penile nerve^8^ has indicated that DGC is a part of the reflex pathways that control the functions of the pelvic viscera^3,14^.

Some structures of the brain stem have a neuroanatomically reciprocal relationship with the lumbosacral spinal cord^15–19^. The DGC in the sacral segment is involved in the central processing of pelvic visceral information and is also associated with nociceptive, analgesia and autonomic function^16^. With excitatory connection to the parasympathetic preganglionic neurons of the lumbosacral spinal cord, the pontine micturition center projections to the inhibitory interneurons of the sacral spinal cord, causing urination by relaxing the external urethral sphincter^20,21^. Functional evidence also indicates that the DGC receives terminations from the afferent fibers of the somatic and viscera^15^.

The nicotinamide adenine dinucleotide phosphate-diaphorase (NADPH-d) reaction is used as a marker to characterize certain neuronal properties and colocalize with nitric oxide synthase (NOS)^22,23^. However, some researchers demonstrate that NADPH-d is not always identical to NOS activity^24–27^. In both central and peripheral nervous systems, only a part of NOS-positive neurons colocalize with NADPH-d histochemistry^24^. The bioactivity of NOS and NADPH-d depends on different cellular locations^28^. Neurons with NADPH-d activity have been shown to exhibit colocalization with several neuropeptides in various brain nuclei^29,30^. At various segmental levels of the spinal cord of the rat, NADPH-d activity is present in a large percentage of visceral afferent neurons in dorsal root ganglia (DRG)^31–35^. In both the rat and cat, NADPH-d is also present in a prominent afferent bundle projecting from LT to the region of the sacral parasympathetic nucleus^34,36,37^. This afferent pathway closely resembles the central projections of afferent neurons innervating the pelvic viscera^38–41^.

Previous studies have shown that NADPH-d-positive neurons and fiber networks are densely stained in the DGC of lumbosacral spinal cord segments in adult animals and may play a specific role in the reflexes of the pelvic organs^42^. A large number of NADPH-d positive neurons in the spinal cord appear to be involved in visceral regulation, innervating most of the pelvic organs, such as the penile tissue^43,44^, internal anal sphincter^45–47^, and lower urinary tract^48,49^. Along with the aging of the spinal cord, the pelvic visceral organs are known to undergo physiological and functional alterations. However, we still know little about the biomarkers and mechanisms involved in the aging process of the spinal cord. Our previous study discovered that neurodegenerations indicated by aging-related NADPH-d positive bodies are specifically present in the lumbosacral spinal cord of aged rats^26^. It could be an onset and progressive marker for pelvic organ dysfunction, which is closely associated with spinal cord aging. Whether these abnormal alterations also occur in the aged lumbosacral spinal cord of other species has not been studied. Therefore, the purpose of the present study was to determine whether aging-related NADPH-d positive abnormalities occur in the lumbosacral spinal cord of aged dogs.

## Results

### NADPH diaphorase activity in the sacral spinal cord of aged dogs

**Figure 1:**
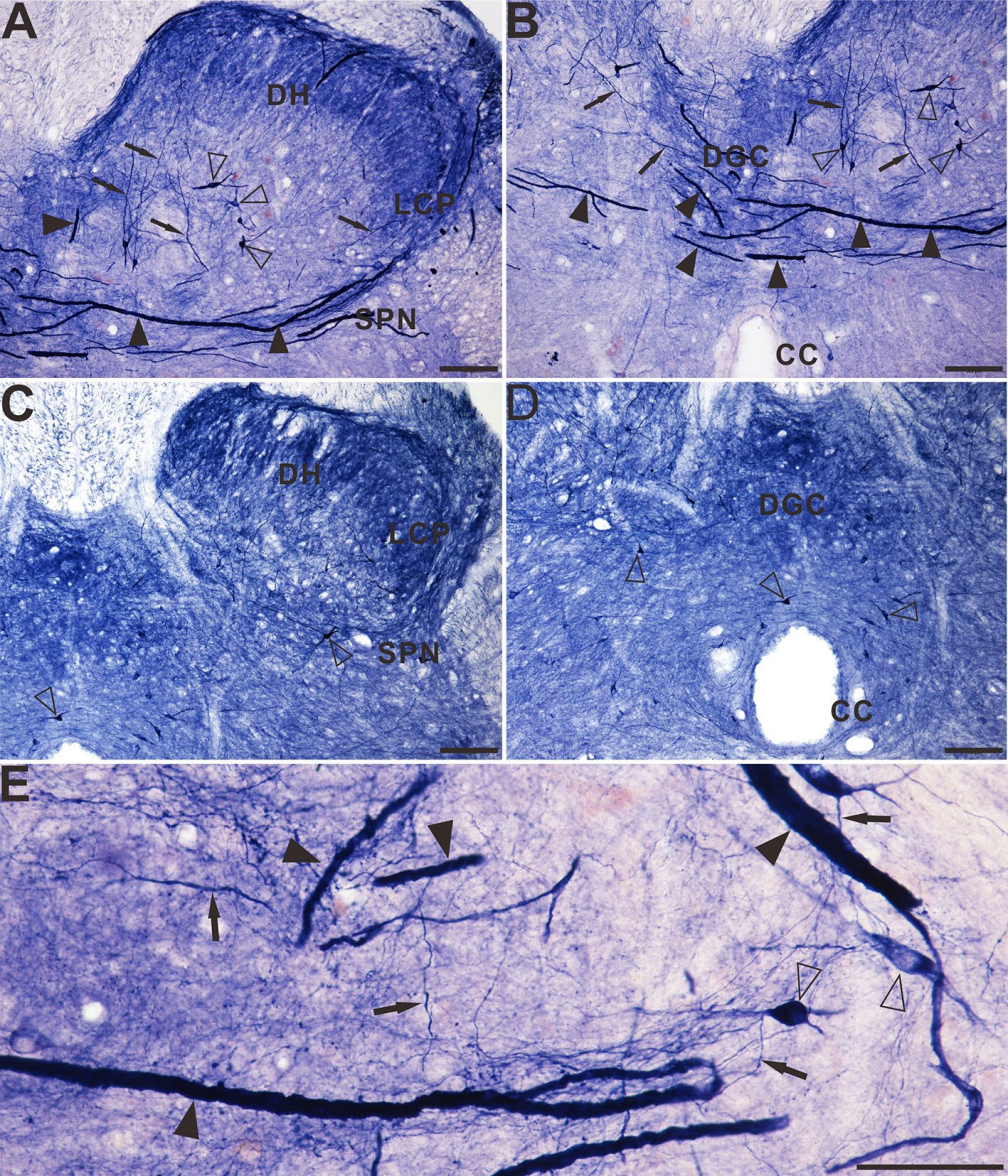
Microphotographs of NADPH-d positive reactivity in aged and young dogs at the sacral (S2 segment) spinal cord. All of the transverse sections are taken at the same levels. Note intense and abnormal NADPH-d positive megaloneurites (black arrowheads) in the LCP (**A**) and DGC (**B**) in the sacral segment of aged dogs compared with young dogs (**C**) and (**D**). The NADPH-d positive megaloneurites in the sacral spinal cord of aged dogs are completely different from the surrounding normal fibers and neurons (**E**). Open arrowheads: NADPH-d positive neurons, black arrows: normal NADPH-d positive neurites, black arrowheads: megaloneurites. Scale bar in (**A**)–(**D**) = 100 μm, in (**E**) = 50 μm.

This study evaluated primarily the dorsal part of the sacral spinal cord, especially the lateral collateral pathway (LCP) of LT, the sacral parasympathetic nucleus, and the DGC because of the distribution of the NADPH-d positivity. In young dogs, NADPH-d positive reactions of fibers and cells were normally detected in the dorsal horn (DH), LT, LCP and DGC, which is consistent with previous discovery^50^. In the dorsal horn and DGC of the sacral spinal cord of aged dogs (Fig. 1A,B), a non-somatic neuronal structure with an expanded diameter was observed that was extremely different from that of the young dogs (Fig. 1C,D), especially in the LCP of the LT (S1–S3). The swelling giant NADPH-d positive alterations were named megaloneurites, a newly coined word, occurred in aged dogs (Fig. 1E). The general location of the NADPH-d positive megaloneurites and selective segmental distribution were related to the central projection of the primary pelvic visceral sensation (black arrowheads in Fig. 1A,B), mostly located dorsal of the spinal cord.

**Figure 2:**
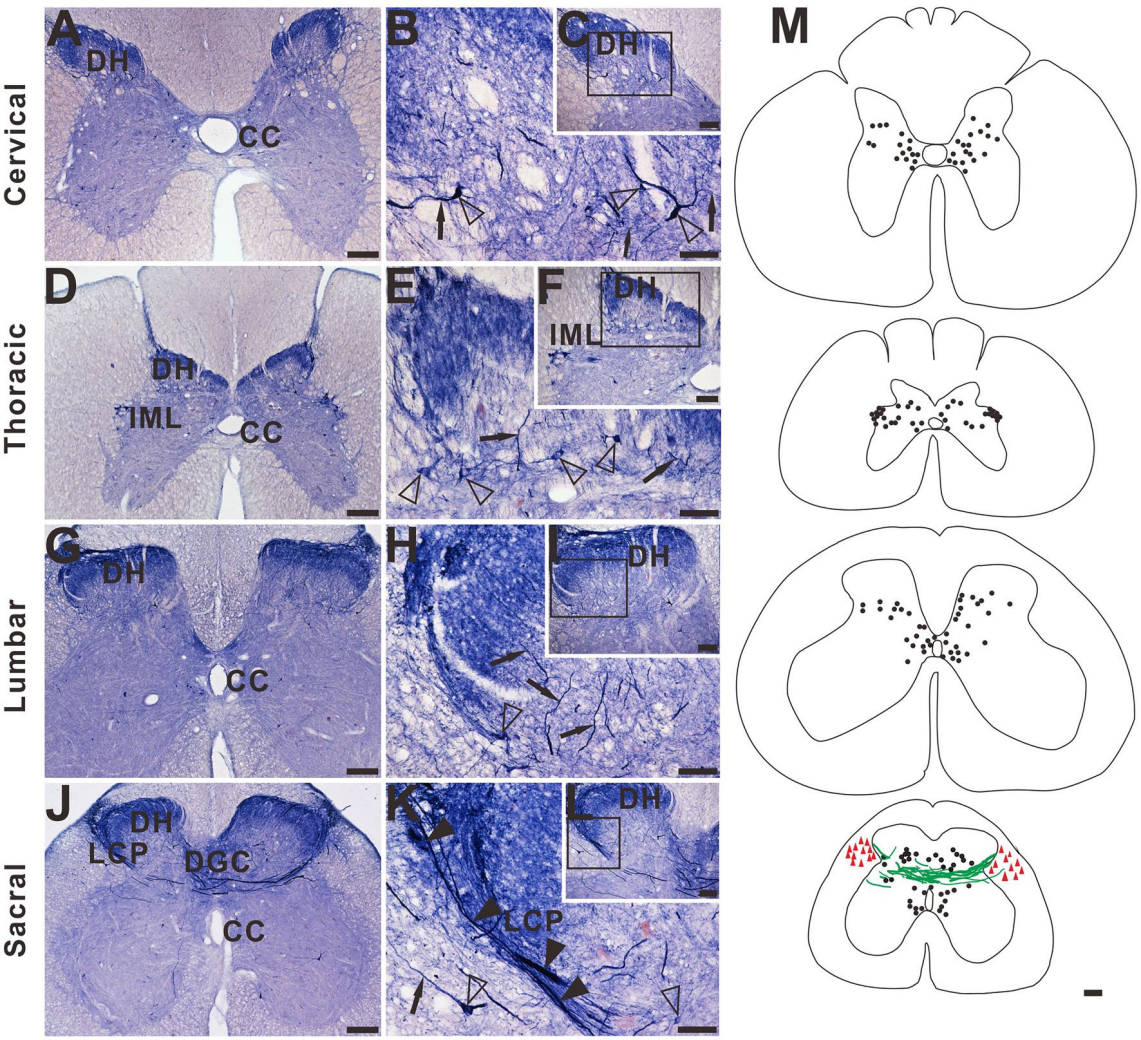
Microphotographs showing the distribution of NADPH-d staining in transverse sections of the aged dog spinal cord at different segmental levels. Few neurons (open arrowheads) and normal fibers (black arrows) staining in the dorsal horn (DH) is present in cervical (**A**), thoracic (**D**) and lumbar (**G**) segments. Intense and abnormal fiber (black arrowheads) staining in the dorsal gray commissural (DGC) and LCP is present in the sacral (**J**) segment. (**B**), (**E**), (**H**), and (**K**) show higher magnifications from insert (**C**), (**F**), (**I**), and (**L**), respectively. (**M**) Schematic diagram of NADPH-d activity taken from the cervical, thoracic, lumbar, and sacral spinal cord segments. Neuronal cell bodies are indicated as filled circles (black) on both sides of each figure. Each filled circle represents one NADPH-d positive neuron. Dense NADPH-d stained megaloneurites are represented by cords (green). The triangle symbols (red) indicate NADPH-d activity in the white matter. NADPH-d stained neurons and fibers from 5 sections are plotted on a drawing of the transverse section of the aged dogs of the spinal cord at indicated segmental levels. Open arrowheads: NADPH-d neurons, black arrows: normal NADPH-d positive neurites, black arrowheads: megaloneurites. Scale bar in (**A**), (**D**), (**G**), (**J**), (**M**) = 200 μm; (**C**), (**F**), (**I**), (**L**) = 100 μm; (**B**), (**E**), (**H**), (**K**) = 50 μm.

Further segmental examination was to demonstrate that the megaloneurites occurred in a specific regional localization. In transverse sections of caudal spinal segments, NADPH-d staining fiber network of dendrites and axon terminals were observed in the superficial dorsal horn (laminae I, II), the DGC, and the dorsal lateral funiculus but not in the ventral horn (Fig. 2A–L). As mentioned above, the most prominent fiber staining in the sacral segments of aged dogs was in LT in laminae I along with the lateral edge of the dorsal horn deeply into the DGC and/or passing across the DGC to the opposite gray matter (Fig. 2J–L). Megaloneurites were selectively detected in the sacral spinal cord (S1–S3) but not in adjacent rostral (L5-L7) or caudal (Cx1-Cx2) segments or in thoracic and cervical segments (Fig. 2A–F,M).

**Figure 3:**
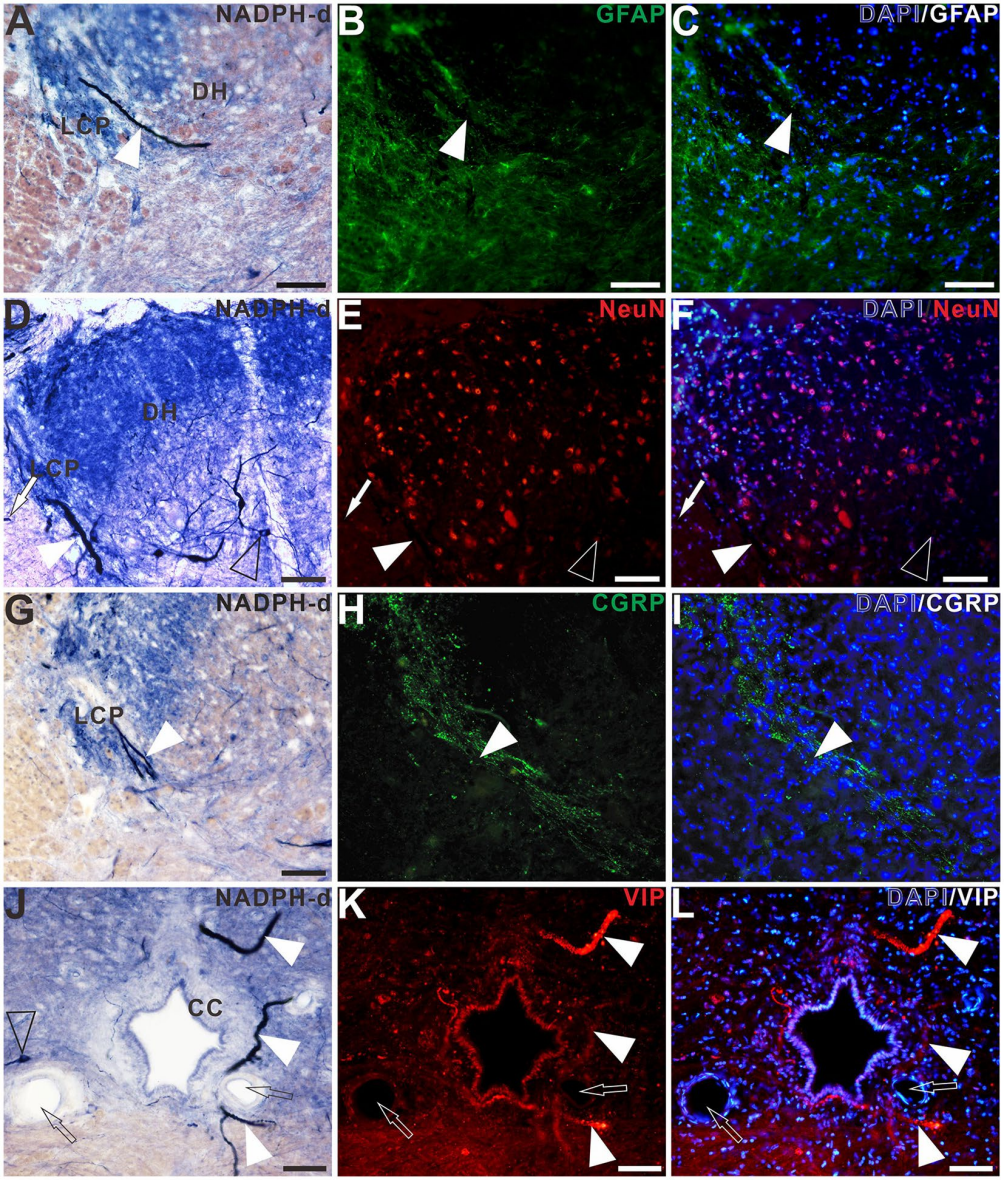
Double-staining of NADPH-d histology combined with GFAP (**A**)–(**C**), NeuN (**D**)–(**F**), CGRP (**G**)–(**I**), VIP (**J**)–(**L**) immunofluorescence in the sacral segment of aged dogs. The NADPH-d positive megaloneurites (white arrowheads) in the LCP are negative for GFAP, NeuN and CGRP immunoreactivity, but positive for the VIP immunoreactivity around the CC. Open arrowheads: NADPH-d positive neurons, white arrowheads: NADPH-d positive megaloneurites, open arrows: vascular structures, white arrows: NADPH-d positive structures in the white matter. Scale bar = 50 μm.

**Figure 4:**
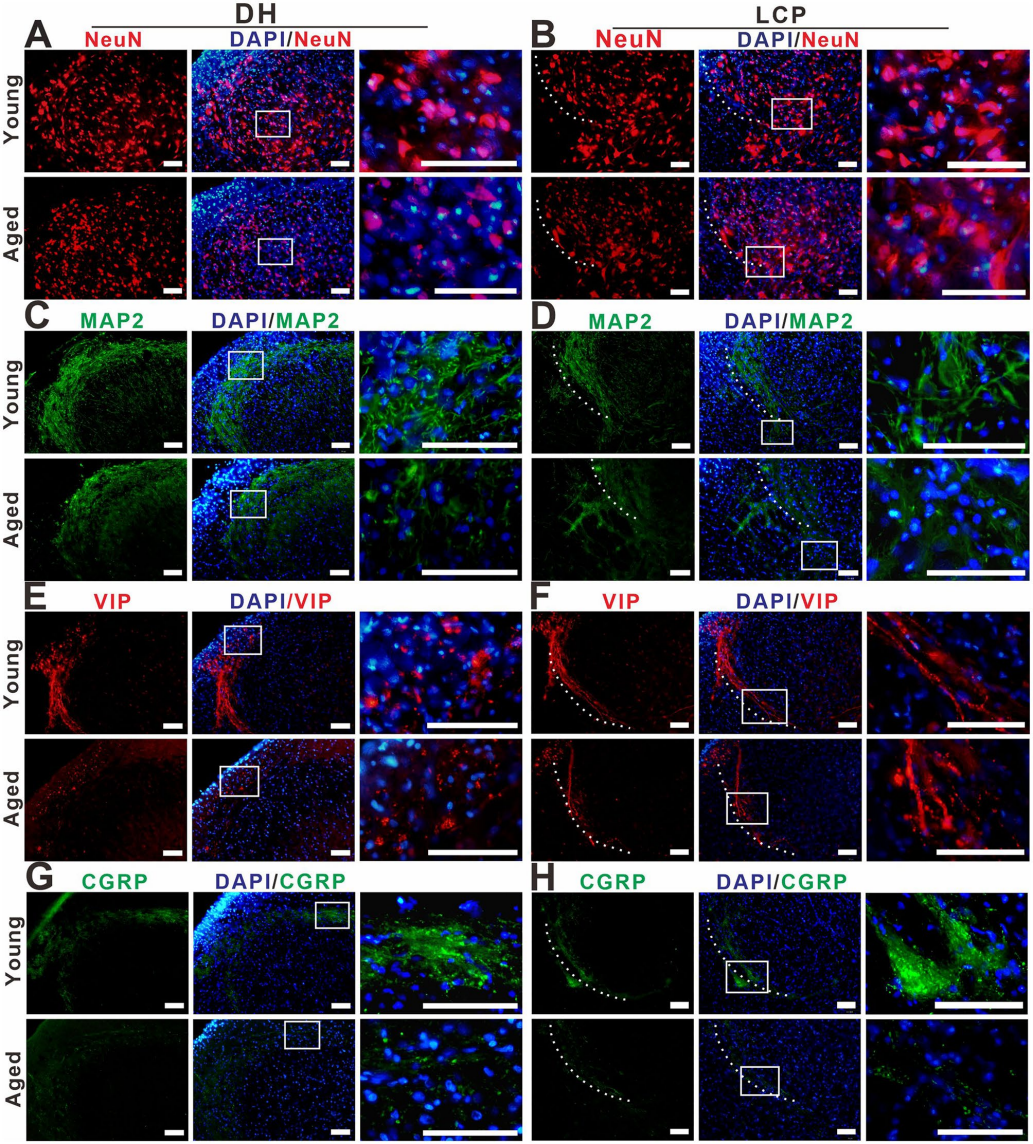
The positivity of NeuN (**A**), (**B**), MAP2 (**C**), (**D**), VIP (**E**), (**F**) and CGRP (**G**), (**H**) immunoreactivity in the DH and LCP of the sacral spinal cord of young and aged dogs. Megaloneurites indicated with VIP positive (E, F). The white dotted lines represent the region of LCP. Scale bar = 50 μm.

**Figure 5:**
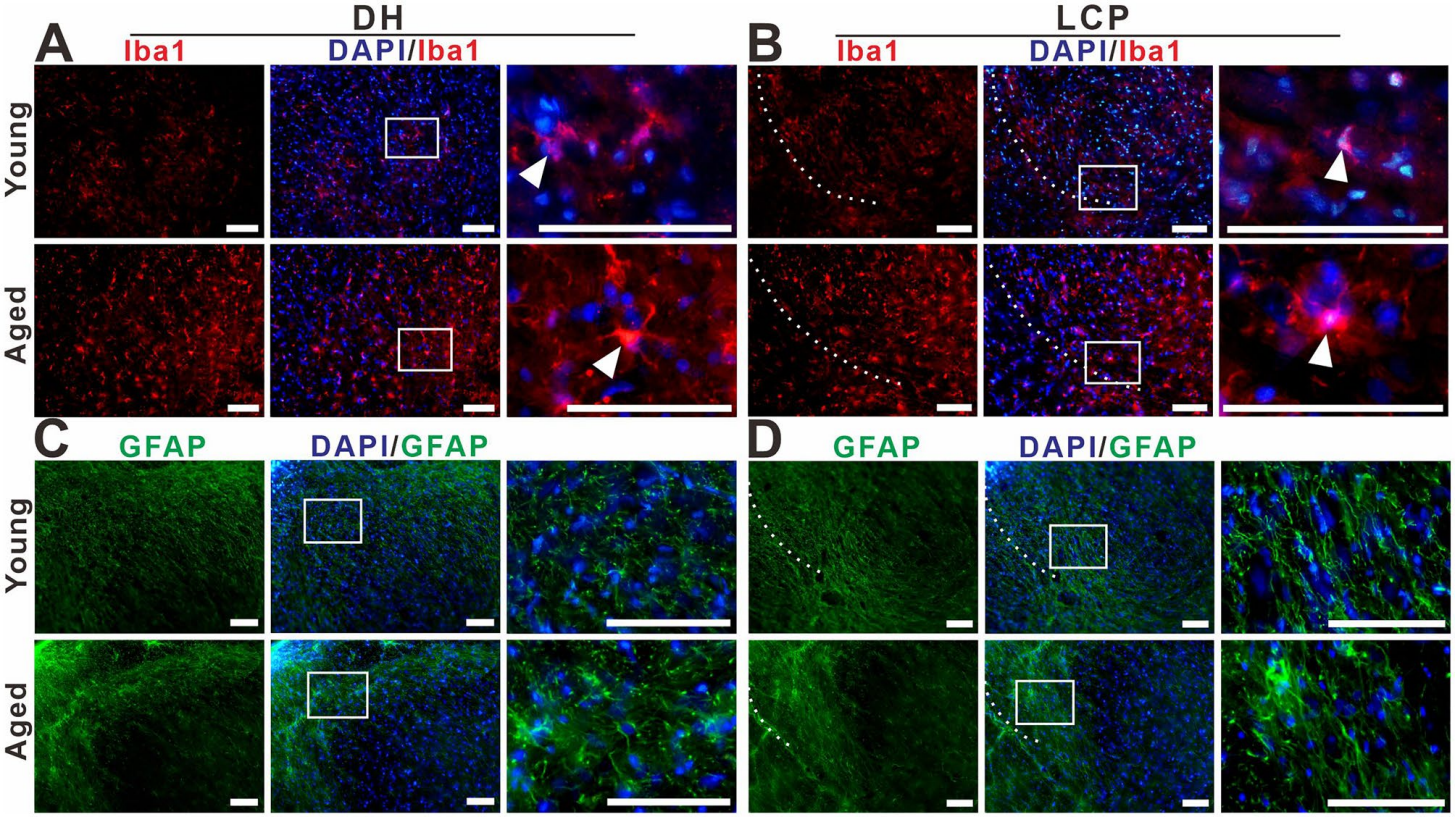
The distribution of Iba1 (**A**), (**B**) and GFAP (**C**), (**D**) immunoreactivity in the DH and LCP of the sacral spinal cord of young and aged dogs. The white dotted lines represent the region of LCP. White arrowheads: microglia. Scale bar = 50 μm.

The double-staining of NADPH-d histochemistry combined with GFAP, NeuN, CGRP, and VIP immunofluorescence were used to characterize megaloneurites, respectively (Fig. 3A–L). It was found that NADPH-d positive megaloneurites were not co-localized with GFAP, NeuN or CGRP immunofluorescence reactions (Fig. 3A–I). It is interesting to discover that NADPH-d positive megaloneurites co-localized with vasoactive intestinal peptide (VIP) immunoreaction (white arrowheads in Fig. 3J–L), LCP, DCN and the dorsal root entry zone (Fig. 4E,F). In comparison to young dogs, a significant decrease in the number of neurons and a dramatic reduction in neurite processes in the dorsal horn of the sacral segments was observed in aged dogs by immunofluorescence of NeuN (Fig. 4A,B), MAP2 (Fig. 4C,D) and CGRP (Fig. 4G,H). These changes might be related to the degeneration of neurites in the aged. In addition, fluorescence expression of Iba1 in the sacral spinal cord of aged dogs was significantly up-regulated (Fig. 5A,B). In the superficial dorsal horn of aged dogs, the expression of fibrous astrocytes with elongated processes and fewer branches was sharply reduced, while the protoplasmic astrocytes with thicker processes and more branches were increased (Fig. 5C,D).

**Figure 6:**
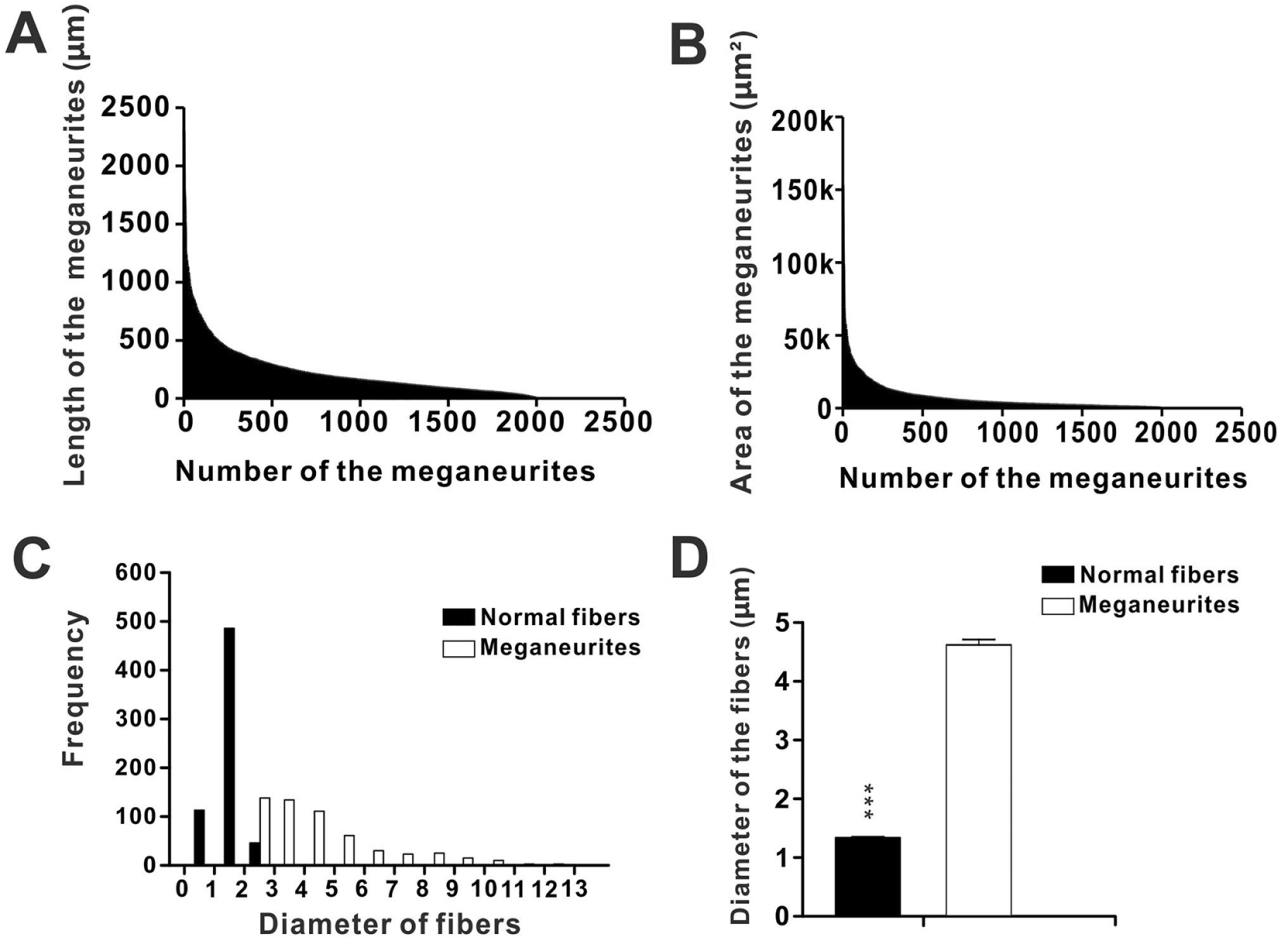
Charts showing length (**A**) and area (**B**) of the NADPH-d positive megaloneurites in the sacral spinal cord of aged dogs (2000 megaloneurites counted). (**C**) Histogram of diameter distribution of NADPH-d positive fibers. (**D**) The diameter of the megaloneurites (500 NADPH-d megaloneurites counted) and normal fibers (500 fibers counted). The k of unit label in (**B**) represents 1000, and asterisks in horizontal bars indicate statistically significant comparisons (***p < 0.001).

Statistical data indicated that the intensely stained NADPH-d megaloneurites ranging between 5 and 2296.4 μm in length identified on transverse sections of thickness 40 μm were regularly seen (Fig. 6A). The area of the megaloneurites ranged between 80 and 153217μm^2^ (Fig. 6B). In the histogram, a large proportion of NADPH-d megaloneurites were between 2.5 and 5 μm in diameter, with a maximum thickness of 10 μm or more, whereas normal fibers were between 1 and 2 μm in diameter (Fig. 6C). The average diameter of the megaloneurites (4.47 ± 0.101 μm) was thicker than that of the normal fibers (1.32 ± 0.017 μm) of aged dogs, and was much thicker than the NADPH-positive fibers of young dogs (1.89 ± 0.017 μm) (Fig. 6D).

**Figure 7:**
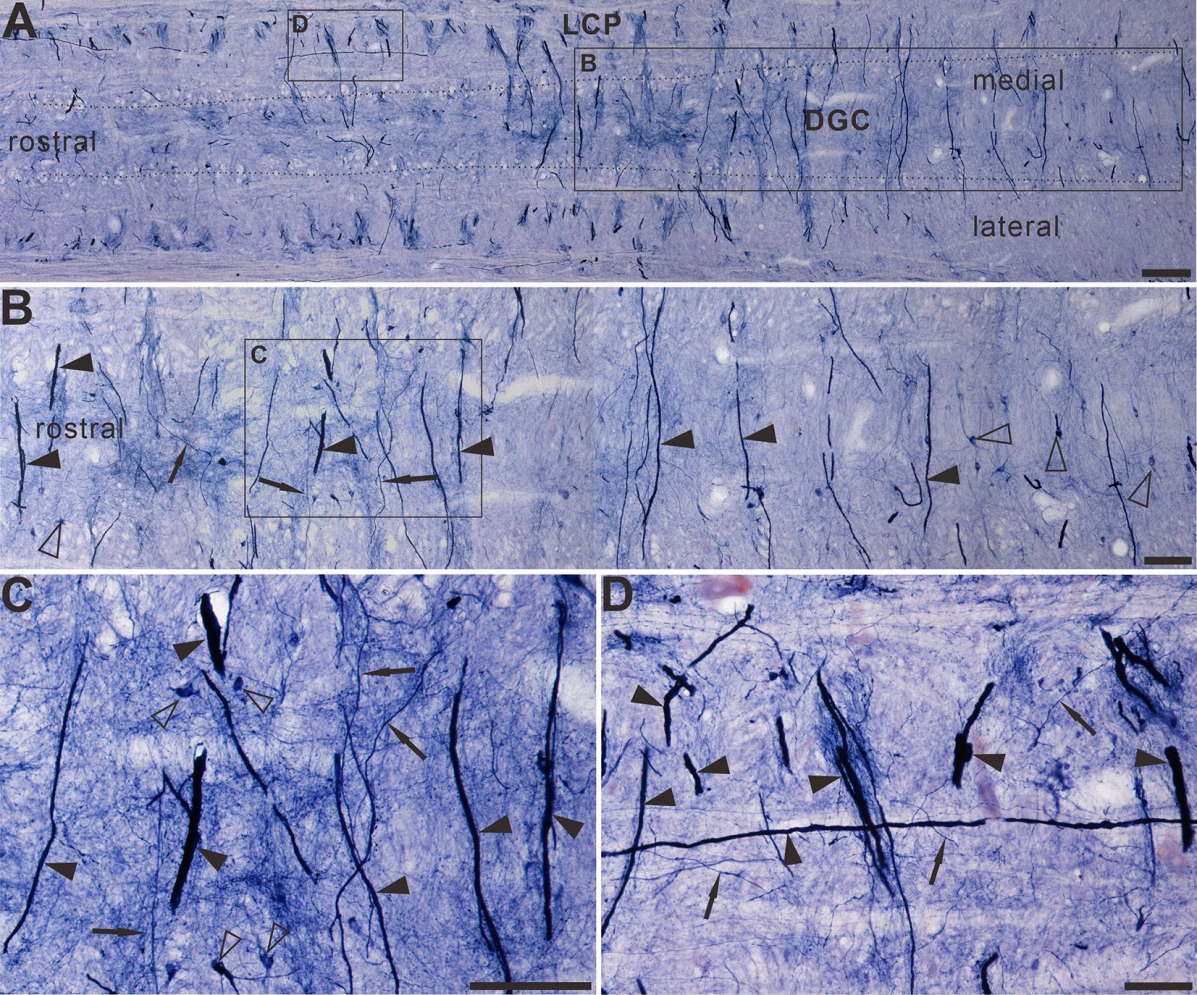
The Megaloneurites in horizontal sections in the DGC in the sacral segment of aged dogs. (**A**) Horizontal sections in the low power microphotograph confirmed that the megaloneurites are organized in a regular interval vertical to the rostrocaudal axis. (**B**) is the magnification of (**A**). (**C**), (**D**) High power microphotographs demonstrated a significant difference between the megaloneurites and normal fibers and neurons. Open arrowheads: NADPH-d neurons, black arrows: normal NADPH-d neurites, black arrowheads: abnormal megaloneurites. Scale bar in (**A**) = 200 μm, in (**B**) = 100 μm, in (**C**), (**D**) = 50 μm.

The horizontal sections of the sacral cord indicated the distribution pattern of megaloneurites (Fig. 7A–D). These spatial arrangements correspond to the megaloneurites detected in transverse sections in Fig. 2J and Fig. 1A,B of the DGC or mediated SPN. In horizontal sections of the sacral cord, megaloneurites were confirmed to be present in the DGC almost vertically along the rostro-caudal axis. Longitudinally oriented megaloneurites occurred more frequently in the LCP of LT and DGC (Fig. 7A). At higher magnifications (Fig. 7C), typical megaloneurites were much larger than regular NADPH-d positive neuronal processes (black arrows). The majority of megaloneurite terminals were branched, with branches having a significantly thinner diameter (5.74 ± 0.26 μm) than that of megaloneurites (12.57 ± 0.66 μm), resulting in a diameter reduction rate of 49%. Individual or clustered megaloneurites (Fig. 7B) were spaced approximately 186.6 ± 5.38 μm apart, as calculated between adjacent midpoints of megaloneurites. It is postulated that megaloneurites in LCP may not be present in every section and may occur intermittently along the rostro-caudal axis.

### NADPH-d alterations around the CC in the aged coccygeal spinal cord

**Figure 8:**
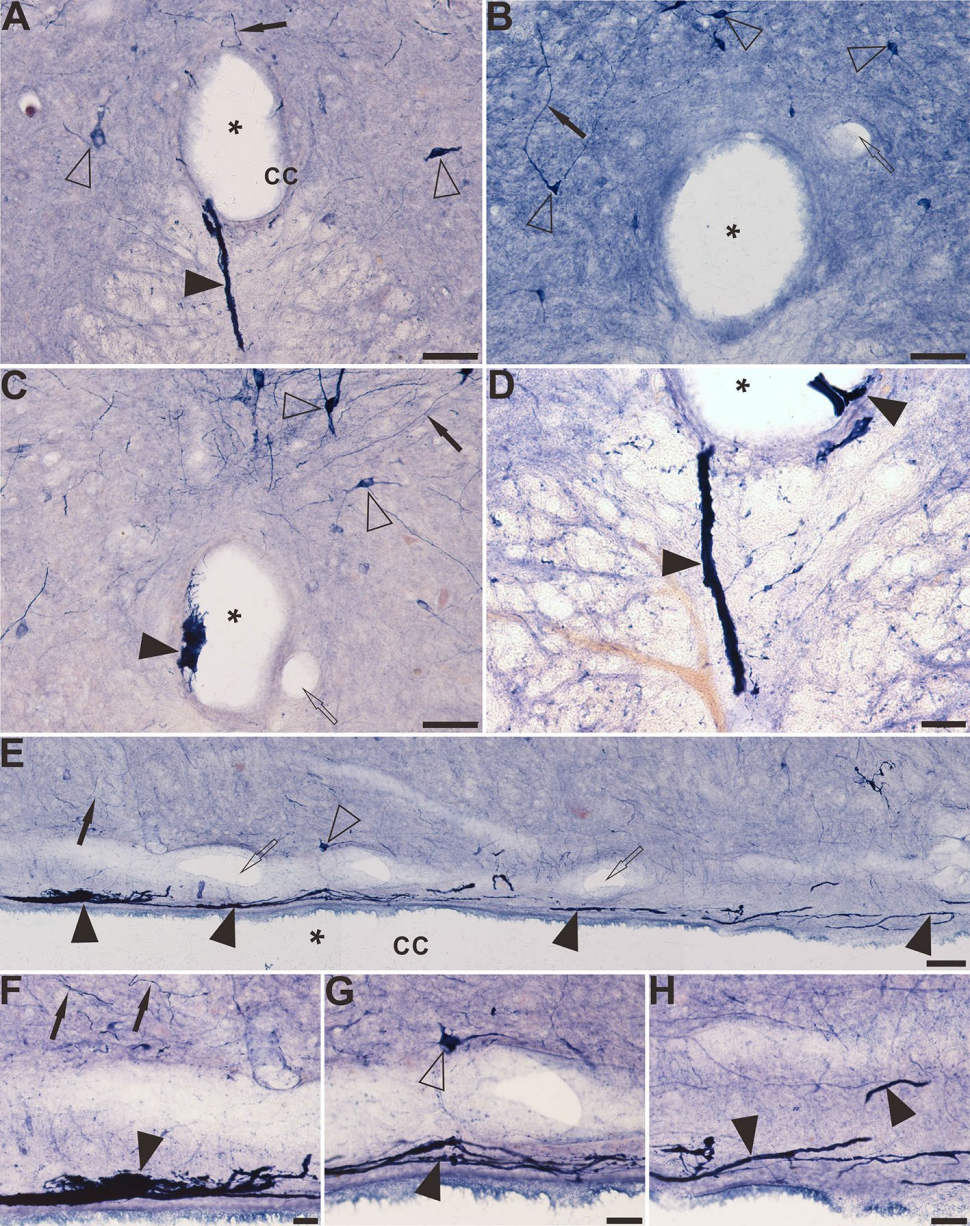
The distribution of NADPH-d positive abnormality in the sacral-coccygeal spinal cord of aged dogs. (**A**), (**C**) and (**D**) demonstrate the location and morphology of the NADPH-d abnormalities on the transverse section of aged dogs. Some positive fibers and structures cross the ependymal cells and reach the lumen of the CC (**A**). There are no aberrant structures in the CC of young dogs (**B**). Note abnormal mass-like or strand-like NADPH-d positive structures (black arrowheads) around the CC of aged dogs. Both intra-CC and extra-CC alterations are detected in (**D**). Megaloneurites indicated ventrally bridging from the CC to the anterior median fissure (**A**) and (**D**). (**E**)–(**H**) shows the location and morphology of the NADPH-d abnormalities along the CC in the horizontal section of aged dogs. Open arrowheads: NADPH-d positive neurons, black arrowheads: NADPH-d positive abnormalities, open arrows: the vascular structures, black arrows: normal NADPH-d positive neurites, the asterisk indicates lumen of CC. Scale bar in (**A**)–(**C**), (**E**) = 50 μm, in (**D**), (**F**)–(**H**) = 20 μm.

In the dorsal horn and DGC of the coccygeal spinal cord, NADPH-d positive megaloneurites are rarely observed. Another type of NADPH-d positive abnormality was observed around the CC of the coccygeal sections (Fig. 8A–D). These abnormalities consisted of three compartments: intra-CC (Fig. 8C), inter-ependyma and extra-CC subdivisions (Fig. 8A,D). In contrast, these profiles were not found in young dogs (Fig. 8B). They also formed a rostro-caudal organization along with the CC in horizontal sections. The intra-CC part was attached to the lumen of the CC while the extra-CC part was located in the sub-central canal. The irregularly-shaped intensely-stained NADPH-d positive abnormalities appeared in a mass-like or strand-like manner around the CC, unlike normal neurons. The mass-like anomalous NADPH-d positive abnormalities had an area on transverse sections ranging from 26,100 to 77.15 μm^2^ and a diameter of 24.76 ± 3.74 μm. The length of these strand-like NADPH-d positive abnormalities was 117.0 ± 8.87 μm. In the horizontal sections (Fig. 8E–H), the NADPH-d abnormalities were distributed around the sub-central canal in a strip shape, and in some instances, these fiber-like structures could be traced along the entire length of the coccygeal section and could extend up to 1400 μm. The positive aberrant structures in intra-CC may represent extracellular matrix with an amorphous profile. As mentioned above, extra-CC structures were located at the sub-ependymal cellular layer as longitudinal fiber-like organizations with anastomoses occurring between ependymal cells. This means that intra-CC and extra-CC NADPH-d positive components were connected with an inter-ependyma subdivision through these anastomoses. We termed this NADPH-d abnormality as aging NADPH-d neuritic hypertrophy around the CC, a variation of megaloneurites.

### NADPH-d activity in the white matter of aged dogs

**Figure 9:**
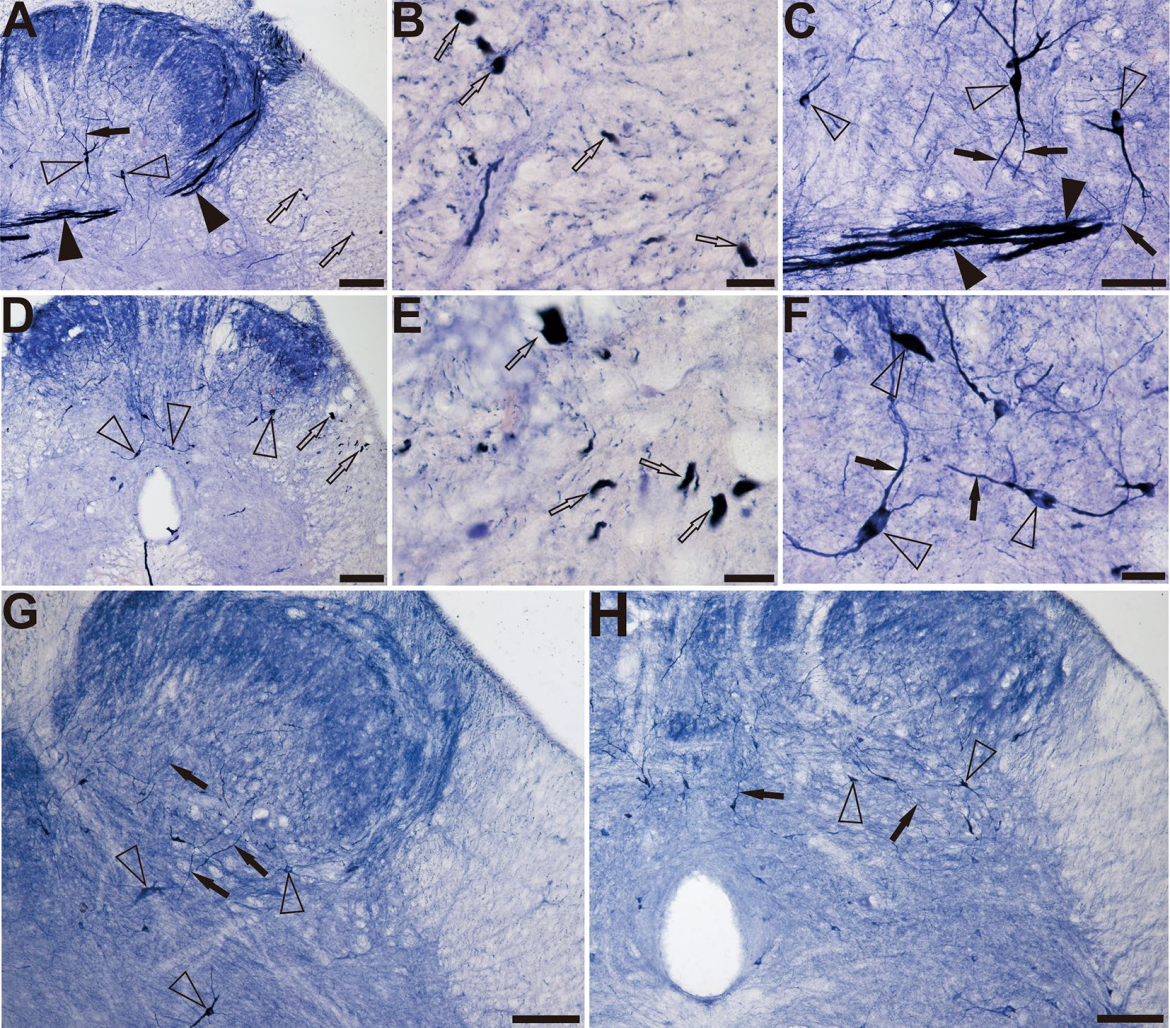
The NADPH-d positive alterations in the white matter of the sacral and coccygeal spinal cord of aged dogs. (**A**)–(**C**) indicate the location and morphology of the megaloneurites in the white matter in the sacral segment of aged dogs and (**D**)–(**F**) represent the coccygeal segment. (**G**) and (**H**) represent the sacral and coccygeal spinal cord of the young dogs respectively. Open arrowheads: NADPH-d positive neurons, open arrows: the alterations of NADPH-d activities detected as transection of megaloneurites (see Fig. 10) in the white matter. Black arrowheads: the NADPH-d megaloneurites, black arrows: the normal NADPH-d positive neuritis. Scale bar in (**A**), (**D**), (**G**), (**H**) = 100 μm, in (**C**), (**F**) = 50 μm, in (**B**), (**E**) = 20 μm.

**Figure 10:**
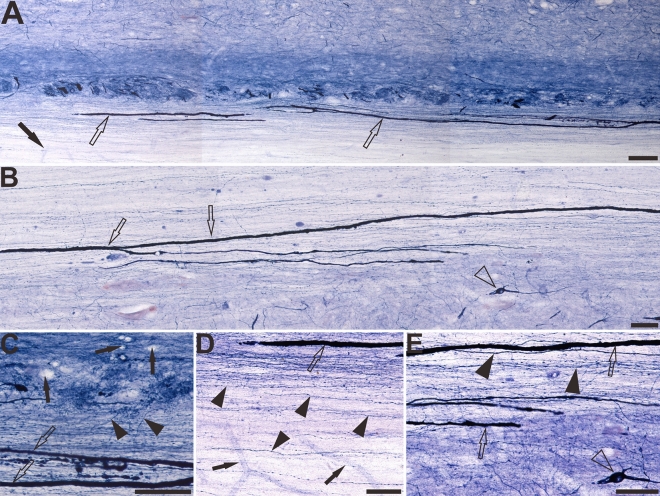
In the horizontal section, the white matter of the lateral fasciculus of the aged dogs. The NADPH-d positive alterations in the white matter of the sacral (**A**), (**C**), (**D**) and coccygeal (**B**), (**E**) spinal cord. Open arrowheads: NADPH-d positive neurons, open arrows: the megaloneurites in the white matter, black arrowheads: normal NADPH-d positive neurites, black arrows: vascular structures. Scale bar in (**A**), (**B**) = 100 μm, in (**C**), (**D**), (**E**) = 50 μm.

In the sacral and coccygeal spinal cord of aged dogs, clearly expressed punctate NADPH-d activity was observed in the white matter (see Fig. 2 schematic drawing of the sacral spinal cord and Fig. 9A–F) compared to young dogs (Fig. 9G,H). This activity was similar to megaloneurites and may have been associated with NADPH-d positive strand-like tracts penetrating deeply into the white matter (Fig. 9B). Considerably higher punctate NADPH-d activity was detected in the lateral portion of the LCP of LT, and the abnormal alterations were extremely different from normal nerve cells and neuroglia cells (Fig. 3). Statistical data indicated that the area of these punctate NADPH-d positive alterations was 43.86 ± 1.098 μm^2^ and the diameter was 6.74 ± 0.07 μm. Horizontal sections of the sacral (Fig. 10A,C,D) and coccygeal (Fig. 10B,E) showed that these punctate NADPH-d alterations were longitudinally-arranged fibrous extending rostrocaudally for 1900–2100 µm in 40-μm-thick sections and were greatly different from normal fiber bundles and vascular structures in white matter (Fig. 10). In addition, numerous NADPH-d fibers and varicosities were evident in LT and in some instances could be traced along the entire length of a section (Fig. 10). We still termed these aged and segmental associated alterations in white matter as megaloneurites.

### NADPH-d activity in the caudal medulla

**Figure 11:**
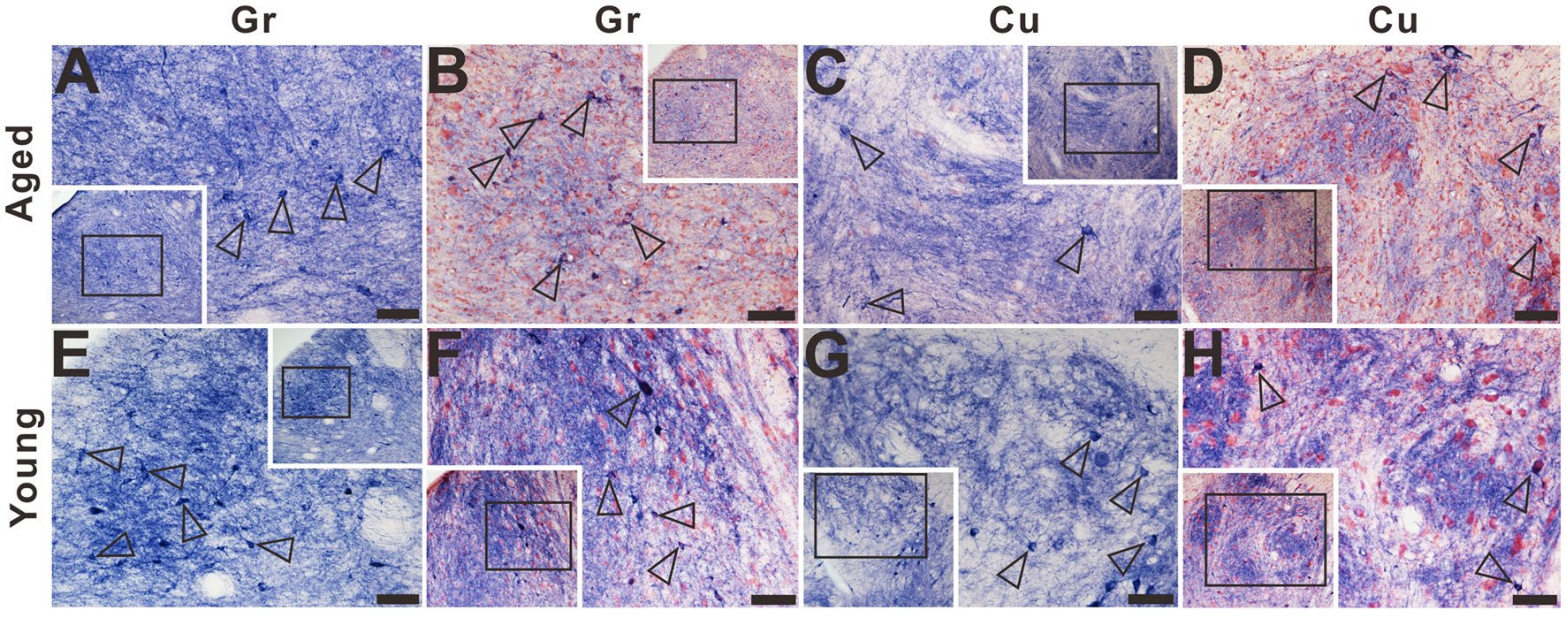
Microphotographs of NADPH-d positive reactivity in the gracile nucleus and cuneate nucleus of aged (**A**)–(**D**) and young (**E**)–(**H**) dogs counterstained with neutral red. (**A**), (**C**), (**E**), and (**G**) show higher magnifications from corresponding insets respectively. No typical NADPH-d positive alterations were detected in the gracile nucleus and cuneate nucleus of aged dogs compared with young dogs. Open arrowheads: NADPH-d positive neurons. Scale bar = 50 μm.

**Figure 12:**
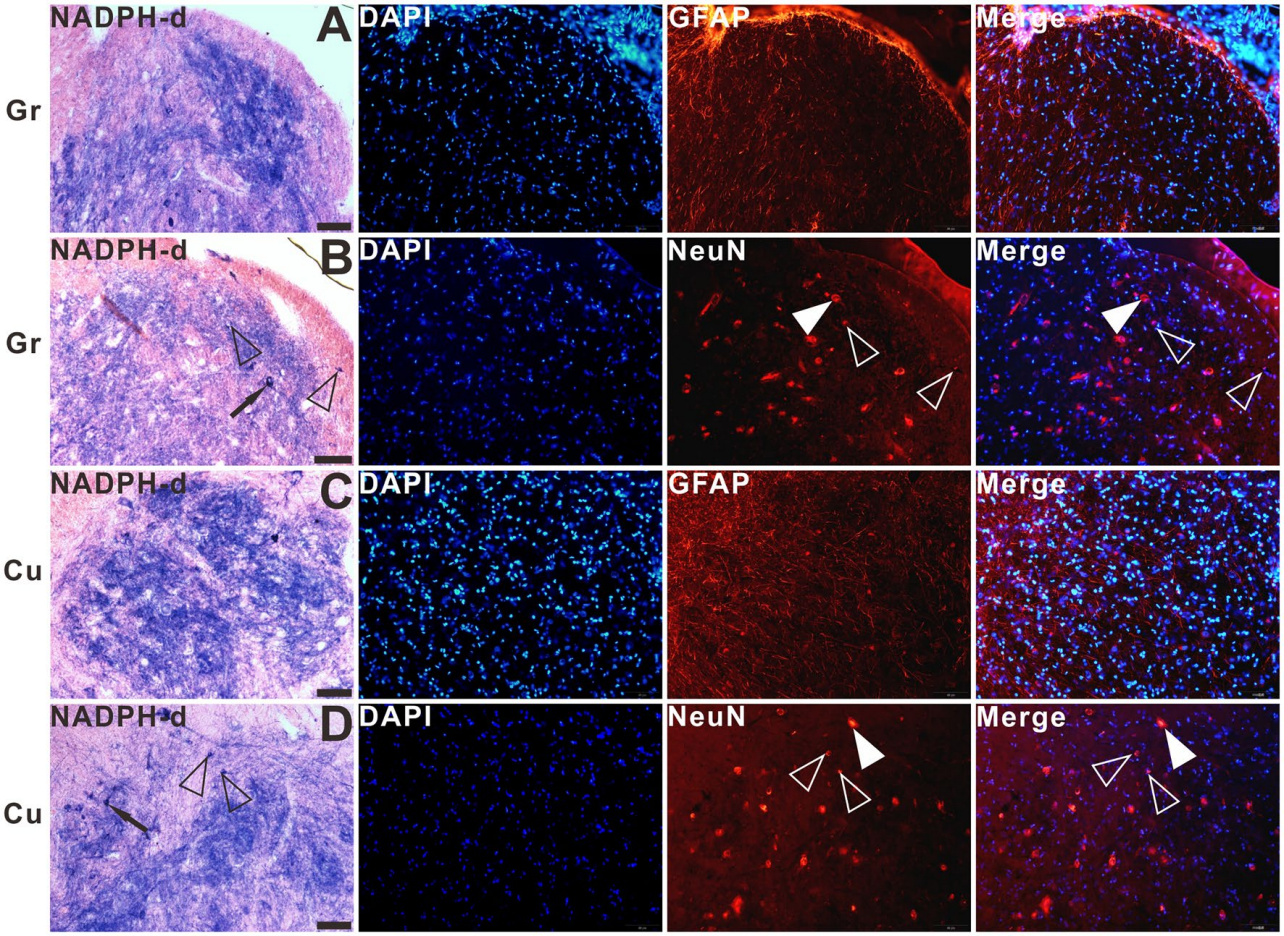
The double-staining of NADPH-d histochemistry combined with GFAP or NeuN immunofluorescence in the gracile nucleus and cuneate nucleus of aged dogs. Open arrowheads: double-labelled neurons, white arrowheads: single-labelled NeuN immunofluorescent neurons, black arrows: single-labelled NADPH-d positive neurons. Scale bar = 50 μm.

In the caudal medulla, primarily in the gracile nucleus and cuneate nucleus (Fig. 11), no NADPH-d positive abnormalities appeared. Small, moderately stained NADPH-d positive neurons were detected both in the gracile nucleus and cuneate nucleus of the aged (Fig. 11A–D) and young dogs (Fig. 11E–H). In the double-stained sections of NADPH-d histochemistry combined with GFAP and NeuN immunofluorescence (Fig. 12), three sub-groups of neurons were identified: single-labelled NADPH-d positive neurons (black arrows in Fig. 12), single-labelled NeuN immunofluorescent neurons (white arrowheads in Fig. 12), and double-labelled neurons (open arrowheads in Fig. 12). As secondary sensory axons project to the thalamus, we also examined the NADPH-d activity of the ventral posterolateral nucleus of the thalamus which receives second-order sensory ascending projections from dorsal column nuclei but found no positive abnormal alterations (data not shown here).

### Changes of NADPH-d positive neurons in the spinal cord at different segments

**Figure 13:**
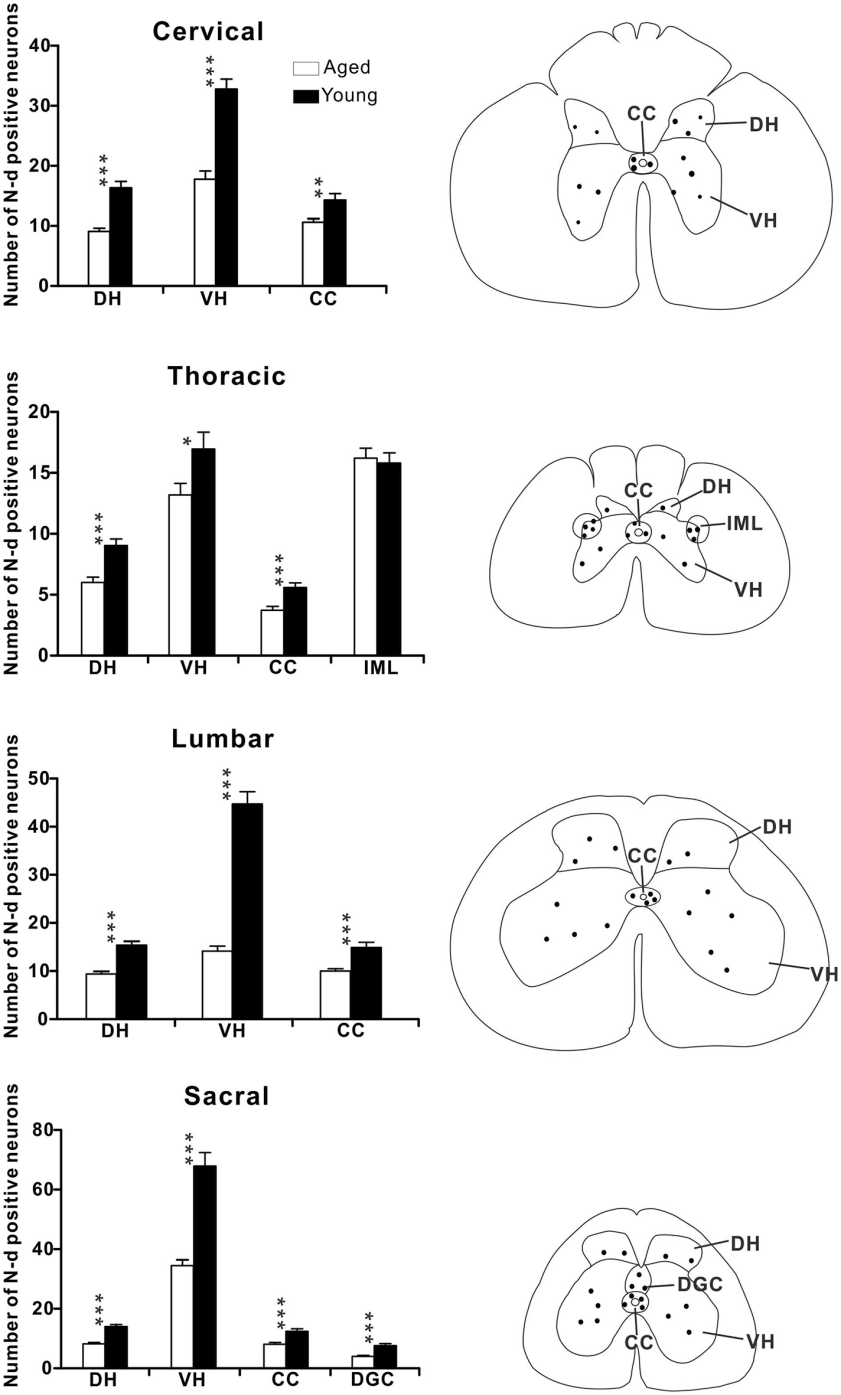
Aging changes of the NADPH-d neurons in the spinal cord. Left panel: histograms showing the number of NADPH-d positive neurons per 40-μm thickness section in sub-regions of the cervical, thoracic, lumbar and segments of the dog’s spinal cord (20 sections examined). Right panel: for specialization areas used in the left panel, representative schematic diagrams based on neurolucida drawings showing the locations of NADPH-d positive neurons (black dots) derived from corresponding sections of the spinal cord. DH: dorsal horn, VH: ventral horn, CC: central canal, IML: intermediolateral cell column, DGC: dorsal gray commissure. *p* < 0.05 was considered statistically significant (*p < 0.05; **p < 0.01; ***p < 0.001).

**Figure 14:**
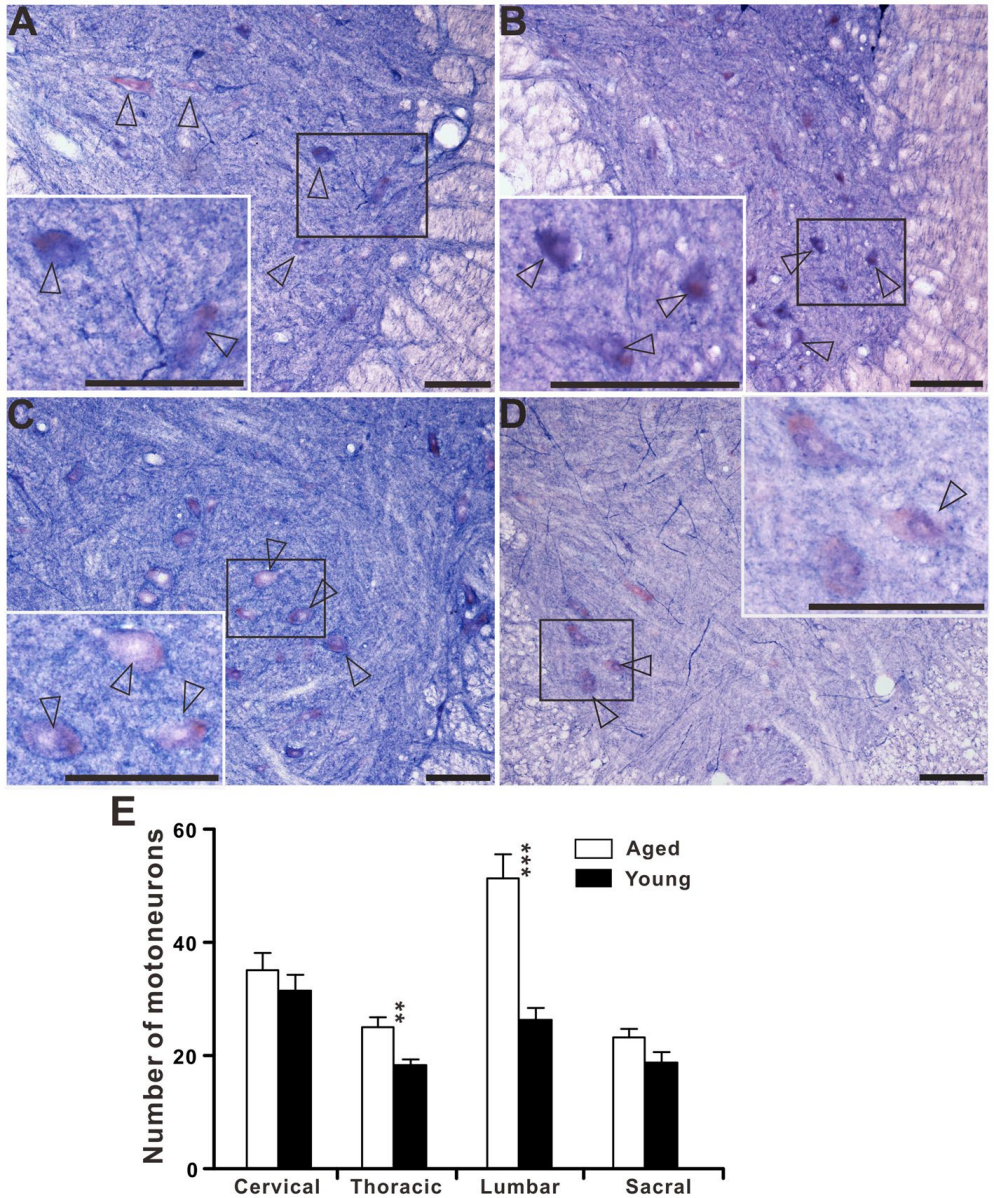
The large, lightly stained motoneurons in the transverse sections in the ventral horn of cervical (**A**), thoracic (**B**), lumbar (**C**) and sacral (**D**) spinal cord of aged dogs. (**E**) Histograms showing the number of motoneurons per 40-μm thickness section in sub-regions of the cervical, thoracic, lumbar and segments of the dog’s spinal cord (20 sections examined). Open arrowheads: large, lightly stained motoneurons. *p* < 0.05 was considered statistically significant (*p < 0.05; **p < 0.01; ***p < 0.001). Scale bar = 100 μm.

We examined changes in NADPH-d positive neurons at different segments in young and aged dogs (Fig. 13). Our data showed that the number of NADPH-d positive neurons was significantly reduced in the dorsal horn, ventral horn^51^, around the CC at all levels in aged dogs compared with young dogs (*p* < 0.05). However, such a decrease was not observed in the IML of the thoracic segment of aged dogs where the number of NADPH-d neurons did not change compared to young dogs (*p* = 0.94). The number of NADPH-d neurons in IML in aged dogs (15.4 ± 1.07 cell profiles/section) was similar to that seen in young dogs (15.5 ± 0.84 cell profiles/section). Accompanied by aging, there was a significant decrease in NADPH-d neurons in the lumbosacral spinal cord of aged dogs (*p* < 0.01). In addition, we observed a group of large, lightly stained motoneurons (Fig. 14) in the ventral horn which were different from intensely stained neurons. Statistical analysis showed that the number of motoneurons in the ventral horn of aged dogs was significantly increased in the thoracic and lumbar spinal cords compared to young dogs (*p* < 0.01), but there was no significant difference between cervical and sacral spinal cords (*p* > 0.05).

### NADPH-d activity in the DRG and dorsal root entry zone

**Figure 15:**
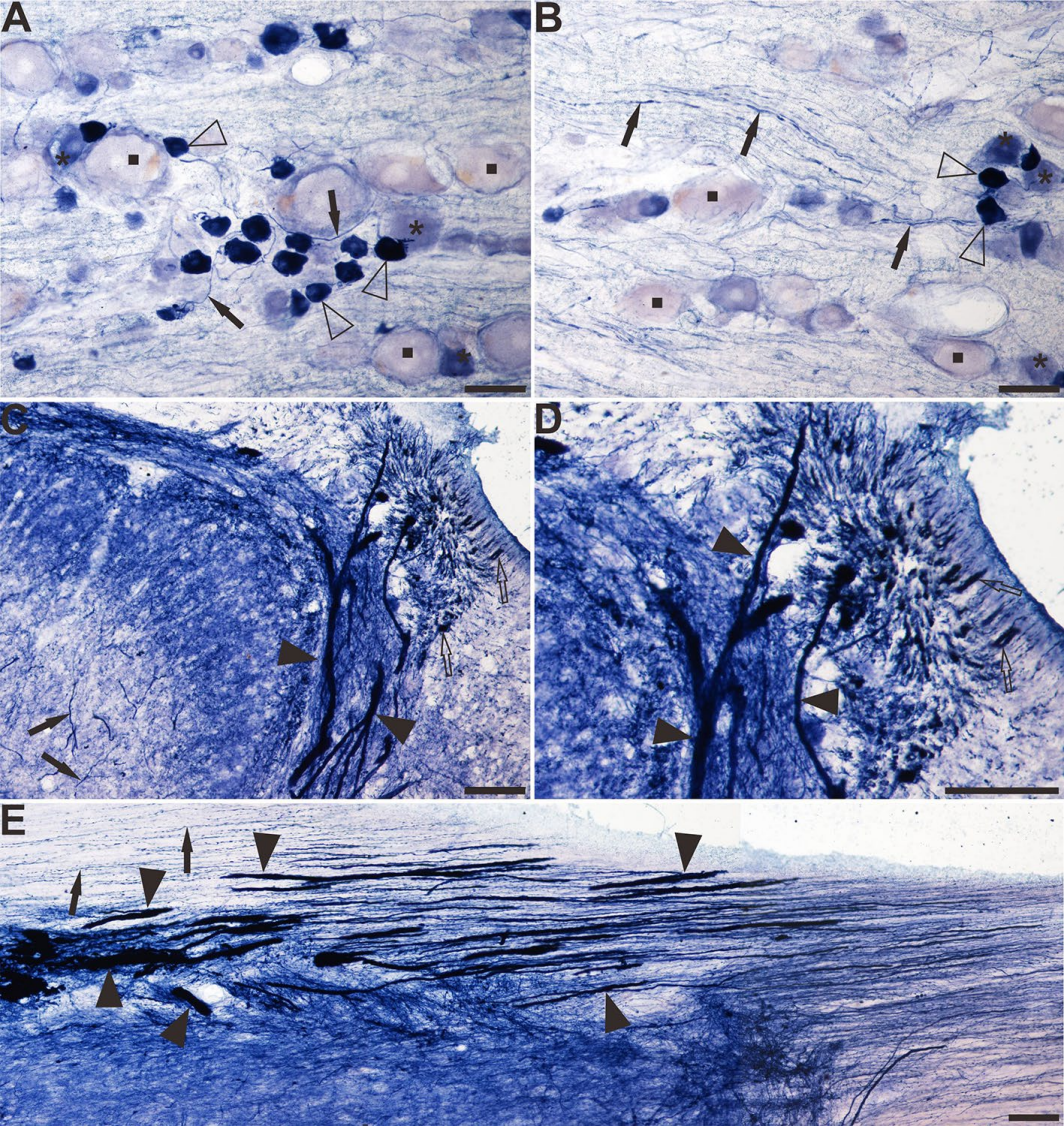
The distribution of NADPH-d positive somata and fibers in the DRG and dorsal root entry zoon of the sacral spinal cord of aged dogs. (**A**), (**B**) A longitudinal section through sacral DRG. Intensely stained NADPH-d-positive cells (open arrows) were observed in the ganglia. (**C**), (**D**) The transverse section in the lateral edge of the dorsal horn at the sacral segment, shows LT filled with NADPH-d activity (open arrows) from its lateral border to beneath the dorsal root (DR). (**E**) Horizontal section at the sacral segment level shows NADPH-d positive neurites and aberrant megaloneurites in the dorsal root entry zone, transitionally continuous with LT. Open arrowheads: intensely stained NADPH-d positive cells, black arrowheads: megaloneurites, open arrows: dots-like NADPH-d activities, black arrows: normal NADPH-d positive neurites, the asterisk indicates lightly stained moderate-size cells, the quadrate indicates unstained large cells. Scale bar in (**A**), (**B**), (**D**), (**E**) = 50 μm, in (**C**) = 100 μm.

In the sacral DRG, cells and fibers exhibited NADPH-d activity (Fig. 15A,B). Small DRG cells exhibited the most intense NADPH-d activity while large cells were either unstained or lightly stained. These results are consistent with previous studies of the DRG^37,52^. In addition, numerous NADPH-d axons were present throughout the ganglia and some exhibited varicosities. The transverse section in the dorsal root entry zone at the sacral segment showed numerous dot-like intensely NADPH-d activities accompanied by NADPH-d megaloneurites in LCP (Fig. 15C,D). In horizontal sections, NADPH-d reaction product was identified in incoming rootlets and within the cord in the dorsal root entry zone associated with the fine fibers continuous with LT (Fig. 15E).

## Discussion

NADPH-d histology can visualize the neuronal processes. NADPH-d reactivity occurs extensively in the spinal cord neurons and sensory pathway^37,42,50,53–58^. Our previous study shows that aging-related NADPH-d neurodegeneration occurs in the lumbosacral spinal cord of aging rats^26^. Although some studies have investigated NADPH-d positivity in the dog’s spinal cord, little report is available on the aging-related changes^50,56,59^.

The focus of this investigation was on aging-related NADPH-d positive alterations in the lumbosacral spinal cord of aged dogs. Our study first revealed a special occurrence of the aging-related NADPH-d positive megaloneurites in the dorsal part of the lumbosacral spinal cord and examined the basic morphology and segmental and laminar distribution. Here we must clarify that the “meganeurite” reported in the previous study is different from the “megaloneurite” we have found. The meganeurite occurs proximal to axonal initial segments of the somas in younger organisms of different locations. The profile of the megaloneurites is the enlargement or swelling of the neuronal processes or neurites, neurodegenerative structures in aged dogs, which is longer than meganeurites^60^. Megaloneurites are non-somatic structures distal to neuronal soma while the meganeurites are shortened proximal enlarged neurites occupying antipodal positions. Regular NADPH-d positive fibers reveal clear puncta and numerous varicosities. Megaloneurites are strongly stained with fewer puncta and varicosities but can be traced for considerable distances in regular existing neuroanatomical structures, especially when confirmed in horizontal sections revealing megaloneurites in dorsal root entry zone, white matter of lateral fasciculus and around central canal. We classified megaloneurites or abnormalities into four major subcategories according to anatomical positions: (1) megaloneurites in gray matter; (2) megaloneurites in white matter; (3) megaloneurites in dorsal entry zone; and (4) aging NADPH-d neuritic hypertrophy around the CC. This specialized aging alteration of neuronal processes is related to a hypertrophy condition in aged dogs.

In this study, the dense hypertrophic megaloneurites extend from the dorsal entry zone and LT through lamina I along the lateral edge of the dorsal horn (LCP) to the region of the sacral parasympathetic nucleus (SPN) and the DGC. The sacral DGC and the LCP receive terminations from the somatic and visceral afferents^9,10,15,38^. Functionally, the sacral spinal cord is known to be associated with bowel bladder, and sexual dysfunction^4–6^. Previous studies have demonstrated^60^ that the sympathetic preganglionic neuron populations that project into the major pelvic ganglion, and the spinal inputs that they receive, exhibit numerous degenerative changes in aged rats (24 months old) which were not seen in the parasympathetic preganglionic neuronal populations. However, the distribution of the megaloneurites overlapped both the efferent and afferent pathways of the autonomic system, which regulates the pelvic organs. Dorsal–ventral rhizotomy eliminates fiber staining in LT and the LCP in both the cat and the rat, but does not alter staining in the dorsal commissure or dorsolateral funiculus^34^. This indicates that fibers staining in LT and the LCP reflect afferent projections.

While the histologically observed segmental distribution of NADPH-d activity in dog is comparable to that described in the spinal cord of other species such as rat^31,53,57,61^, and cat^37,52^, striking differences are noted in the LCP of LT and DGC mainly in the lower lumbar and sacral segments. In addition, the sympathetic autonomic nucleus (IML) in the rostral lumbar segments exhibits prominent NADPH-d cellular staining whereas the parasympathetic nucleus (SPN) in the sacral segments is not well stained^37^. One major difference between NADPH-d staining in rats and dogs is noted in fiber in the LT and the LCP on the lateral margin of dorsal horn in the sacral spinal cord. In dogs, a prominent group of NADPH-d positive fibers travel rostrocaudally in LT and send projections dorsoventrally along the lateral edge of the dorsal horn into base of the dorsal horn where they turn medially. Abnormal NADPH-d fiber bundles or megaloneurites were not revealed in the aged rats. Instead, spherical aging-related NADPH-d positive bodies appeared in sacral DGC and dorsal horn^26^. Another prominent difference in NADPH-d activity between dogs and rats was lack of cell staining in the region of SPN for dogs. In rats, a large percentage of preganglionic neurons in the region of SPN is stained while only a few scattered cells are NADPH-d positive for dogs. No enlarged fibers were reported for aged rat white matter nor were any aberrant NADPH-d changes found around aged rat CC.

The megaloneurites were confirmedly detected in horizontal sections as discontinuous fiber bands extending mostly in the transverse plane, and the fibers exhibited a periodicity with an interval of approximately 180 μm. Based on these profiles, we suggest that they are a part of the neurites in the lumbosacral dorsal spinal cord. The primary question is whether aging-related megaloneurites arise from neuroglia or blood vessels. On one hand, the NeuN immunofluorescence failed to label the megaloneurites. NeuN immunoreactivity could be detected in nuclei, perikarya, and some proximal neuronal processes^62^. on the other hand, the aging-related megaloneurites in aged dogs were clearly distinguished from the glia and endothelium, as the size of typical ones was bigger than regular glia and significantly different from vascular structures found under light microscopy^26^. Interestingly, we found that megaloneurites co-localized with vasoactive intestinal peptide (VIP) immunofluorescence reactivity. VIP is a basic 28-amino acid peptide which is released in the central and peripheral nervous system to support neuronal survival under physiological and pathological conditions^63,64^. VIP is involved in the information transmission and physiological regulation of many functions of the organism, and is widely distributed in the circulatory, immune, reproductive, and digestive systems, as well as in the central and peripheral nervous systems^65^. Meaningfully, VIP can be considered as a marker of parasympathetic nerves, and branches of the pelvic plexus innervating the prostate gland exhibit VIP immunoreactivity^66^. Studies have shown that VIP immunoreactivity (IR) plays an important role in efferent and afferent pathways of the autonomic nervous system that innervates the pelvic organs^67–70^, primary afferent neuropathy induced upregulation of VIP immunoreactivity in the dorsal horn of the spinal cord^71–73^. Previous studies have also shown the distinctive distribution of VIP-containing fibers and terminals in the lumbosacral spinal cord, which may play an important role in the spinal control of urogenital function in humans^74^. NADPH-d positive fibers were similar to the distribution and projection of VIP immunoreactivity in the LCP region of the sacral spinal cord in adult male dogs^50^, which is consistent with our findings. The distribution of megaloneurites in the lumbosacral spinal cord of aged dogs overlapped with the VIP immunoreactivity in our study. It indicated that megaloneurites may affect sensory afferent pathways in the autonomic nervous system of aged dogs compared to young dogs, which contributes to pelvic visceral dysfunction in the aging condition. Further studies are needed to understand the co-localization of megaloneurites and VIP in the role of neurodegenerations in the aging spinal cord to provide a basis for developing VIP-targeted therapies for the treatment of aging-related pelvic organ dysfunction.

Analysis of horizontal sections of aged dogs showed that megaloneurites occurred in the dorsal root entry zone, with the diameter gradually enlarging from distal to proximal in the dorsal root entry zone. The interface between the peripheral nervous system and CNS in the spinal cord and brain stem is considered transitional zone^75^. The enlarged neurites were not found in distal rootlets and DRGs for aged animals. Compared to other segments of spinal cords, the megaloneurites occurred in caudal lumber, sacral and rostral coccygeal in aged dogs. All these segmental selective malformed structures demonstrate that the caudal of the spinal cord especially sacral segments related to adverse conditions more vulnerable to aging alterations.

Neuropathological studies have shown that NADPH-d staining could be used to reveal the neuronal terminal-pathy of aged conditions^76^ as well as neurodegenerative animal models^77^. In our unpublished study in aged rats, aging-related NADPH-d neurodegeneration was detected in the gracile nucleus, which is similarly derived from the aging alterations in the lumbosacral spinal cord^26^. The inputs of primary sensory neurons of DRG could form axon bifurcation in the spinal cord. The bifurcating collaterals can terminate in corresponding spinal segments and ascend to dorsal column nuclei respectively. Besides somatic sensory inputs, dorsal column nuclei also receive ascending visceral sensory information^78^. The NADPH-d neurodegenerations in the lumbosacral spinal cord and gracile nucleus were postulated for dying back in aged rats^79,80^. We did not find significant NADPH-d neuritic alterations in the gracile nucleus and DRG for aged dogs.

Different to rats, concomitant with prominent megaloneurites or anomalous structures were found around CC for aged dogs. Tang et al. reported that NADPH-d positive nerve fibers form the subependymal plexus and occur to traverse ependyma to run internally along CC lumen^81^. NADPH-d ependymal traverse neurons could work as cerebrospinal fluid (CSF)-contacting neurons. Bringing together our findings, aging NADPH-d neuritic hypertrophy around CC was postulated as neuritic dilation of neuronal fiber crossed ependyma forming one kind of neurodegeneration. CSF-contacting neurons function as pH sensors and mechanoreceptors^82^. Depleting CSF-contacting neurons by neurotoxicity does not cause vital status^83^. Megaloneurites and similarity around CC may produce localized disturbance and could then block axonal transport to give rise to morphological alterations identical to denervation.

The number of NADPH-positive neurons was significantly reduced in the spinal cord caused by aging, but in the thoracic spinal cord, the number of NADPH-d positive neurons in the IML did not change. We did not find the NADPH-d positive neurons in the SPN, but the number of NADPH-d positive neurons in the DGC decreased in the aged dogs. The number of sympathetic neurons remained relatively stable. We believe that the sympathetic neurons are important for vital organs. While the neurons innervating pelvic organs may be more vulnerable to aging deterioration.

NADPH-d activity in neurons and fibers in the DRG provides some support for the proposal that NADPH-d reactivity is present in pelvic visceral afferent pathways. NADPH-d staining in DRG cells occurs with different intensities ranging from intense to very light. Intensely stained NADPH-d DRG cells, like visceral afferent and dorsal root ganglion cells, are among the smallest cells in the ganglia^37,84^. This raises the possibility that NADPH-d fiber bundle in LCP might represent central projections of small cells. The pelvic visceral afferents enter the spinal dorsal horn at dorsal root entry zone and continue with LT. Many previous studies have shown that patients with nerve injury impaired sensory afferents enter posterolateral aspect of the spinal cord through dorsal root entry zone and dorsal root entry zone lesions can alleviate neuropathic pain^85,86^.

Our finding is regarding selective neuronal vulnerability attributed to regional, cell type and aging. Although megaloneurites and similarity alterations selectively occurred in sacral spinal cord for aged dogs it is hardly determined that subpopulations of NADPH-d neuronal structures are of segmental variation. Cellular senescence and aging neurodegeneration may determine other intrinsic vulnerabilities of the sacral spinal cord^87^. We do not know if other biochemical properties of similar enlarged fibers existed. Further experiments are required to prove the hypothesis. In general, we found two kinds of aging changes in the spinal cord for aged dogs: non-specific and specific segmental alteration. The number of NADPH-d neurons throughout the spinal cord of aged dogs was markedly reduced without significant segmental changes, providing evidence for pan-neurodegenerative changes, however, megaloneurites specifically occurred in the caudal spinal cord for aged dogs implicating aging dysfunction of urogenital organs.

In this study, we explored the morphological changes that appear in the aged spinal cord by histochemical methods and sought to discover biomarkers that could indicate spinal cord aging. The results indicated that megaloneurite, an aging-related specific neurodegenerative change in the lumbosacral spinal cord of aged dogs, which appeared in the aging process of the spinal cord, might contribute to pelvic visceral dysfunction in the aging condition. The finding of megaloneurites provides a possible reference for future exploration of the biomarkers of spinal cord aging and the development of interventions. However, there are some limitations of this study. On the one hand, we have only reported the results in aged rats and dogs so far, and it is necessary to validate this finding in more species, for which we have also validated the results in non-human primates. On the other hand, we only investigated the morphological changes in the aged spinal cord, without further revealing the pathological and cellular molecular characteristics of megaloneurites. The application of proteomics in future studies will facilitate further explore the characteristics of megaloneurites, which may provide possible intervention targets for delaying spinal cord aging and preventing age-related co-morbidities.

## Conclusion

In summary, this study reveals a major new finding: NADPH-d positive megaloneurites colocalize with VIP in the aged lumbosacral spinal cord but not in young dogs. Megaloneurites may represent swelling of transganglionic fibers located in various regions of the sacral spinal cord including the dorsal root entry zone, lateral dorsal marginal to the dorsal horn, SPN, DGC and white matter as well as CC. These megaloneurites are considered a specific aging marker and indicative of age-associated progressive deterioration and malformed structure.

## Materials and methods

### Animal and tissue preparation

Young (1.59 ± 0.09 years old, n = 6) and aged (11.17 ± 0.68 years old, n = 6) female dogs (Canis lupus familiaris), weighing 5–15 kg, were used in our experiments. These animals did not show any neurological deficits before experiments and were humanely euthanized. The study is reported in accordance with ARRIVE guidelines (https://arriveguidelines.org). All experimental procedures followed the International Guiding Principles for Biomedical Research Involving Animals and the Laboratory Animal-Guideline for ethical review of animal welfare (GB/T 35,892–2018) and were approved by the Ethics Committee in Animal and Human Experimentation of the Jinzhou Medical University (IACUC Number: 2014JCATBH0E-NNSF-0004).

The animals were euthanized with sodium pentobarbital (Laboratory Animal-Guidelines for euthanasia, GB/T 39,760–2018, 80 mg/kg i.v.), perfused transcardially with saline followed by freshly prepared 4% paraformaldehyde in a 0.1 M phosphate-buffered saline (PBS, pH 7.4). Following perfusion fixation, the spinal cords and brains were rapidly dissected out and placed in 25% sucrose for 48 h.

The spinal cords from the cervical to coccygeal segments and gracile nucleus in the medulla oblongata as well as the thalamus were cut transversely into one-in-three series of 40 μm sections on a cryostat. To visualize the rostrocaudal orientation of the NADPH-d positivity, horizontal sections (40 μm) of the spinal cords of the aged dogs were also performed.

### NADPH diaphorase histochemistry

Staining was performed using free-floating sections^22^. Most of the spinal cord sections from the young and aged dogs were stained and examined by NADPH-d histochemistry, with incubation in 0.1 M PBS, 0.3% Triton X-100 containing 1.0 mM reduced-NADPH (Sigma, St. Louis, MO, USA) and 0.2 mM nitro blue tetrazolium (NBT, Sigma), at 37 °C for 2–3 h. Sections were monitored every 30 min to avoid overstaining. The reaction was stopped by washing the sections with the phosphate-buffered saline (PBS, 0.1 M).

### Double immunofluorescence staining

Some sections were processed by double-staining with NADPH-d histochemistry and NeuN, CGRP, VIP or GFAP immunofluorescence and single-staining with NeuN, CGRP, VIP, MAP2, Iba1 or GFAP immunofluorescence, respectively. The sections were collected in PBS in 24-well plates and processed for free-floating immunofluorescence using primary polyclonal antibodies that label neurons (NeuN, mouse; 1:1000, Millipore MAB377, Merck Millipore), reactive astrocytes (GFAP, mouse; 1:1000, Sigma, USA), microtubule associated protein 2 (MAP2, mouse; 1:200, Novus Biologicals, Littleton, CO, USA ), calcitonin gene-related peptide (CGRP, mouse; 1:100, Sigma, USA), vasoactive intestinal peptide (VIP, rabbit, 1:1000 Sigma, USA), microglia (Iba1, rabbit; 1:1000, Wako Chemicals, Japan). Sections were incubated for 1 h at room temperature in blocking solution (0.05 M PBS) at pH 7.4 with 1% BSA. The primary antibody was diluted in PBS containing 1% BSA and applied to the sections for 24 h at 4 °C. In each immunofluorescence testing, a few sections were incubated without primary antibody, as a negative control. The sections were then washed several times with PBS. Fluorescent-conjugated secondary antibodies (IgG anti-mouse Cy3 conjugated [1:2000, Jackson], Goat anti-Rabbit IgG (H + L), Alexa Fluor 594 [1:800, Life] and Goat anti-Mouse IgG (H + L), Alexa Fluor 488 [1:800, Life]), were diluted in PBS and applied to the sections for 1 h at 37 °C in the dark. Finally, after several washes with PBS, the sections were incubated with DAPI for 10 min. The sections were placed on slides and cover-slipped. For controls of immunofluorescence staining, the primary antibodies were omitted or replaced with the same amount of normal serum from the same species while doing the same specific labelling with the normal procedure of immunofluorescence staining. No specified staining was detected in the immunostaining control experiments.

### Measurement of neurons and fibers

Images were captured with a DP80 camera in an Olympus BX53 microscope (Olympus, Japan). Sections were observed under the light microscope and 20 sections from all spinal cord levels in each animal were quantitated using Olympus image analysis software (Cellsens Standard, Olympus). The numbers of NADPH-d neurons were counted on both sides of the spinal cord, on each section of each animal. The diameter of 500 NADPH-d megaloneurites and normal fibers and the length and area of 2000 NADPH-d megaloneurites were also assistantly measured with Neurolucida 360 (MBF Bioscience, Inc, USA).

### Statistics and figure edition

All data are expressed as the mean ± SEM and *p* < 0.05 were regarded as statistically significant. Statistical analyses were performed using GraphPad Prism 9.0 (GraphPad Software, La Jolla, CA). Differences between young and aged dogs of NADPH-d positive neurons in sub-regions of the cervical, thoracic, lumbar and sacral segments were analyzed using unpaired t-tests.

## Data Availability

The data used to support the findings of this study are available from the corresponding author upon request. The original manuscript as preprint has been posted to BioRxiv (bioRxiv 483,990; doi: https://doi.org/10.1101/483990).
